# Inhibition of sodium glucose cotransporter 2 (SGLT2) delays liver fibrosis in a medaka model of nonalcoholic steatohepatitis (NASH)

**DOI:** 10.1002/2211-5463.12598

**Published:** 2019-02-15

**Authors:** Ryo Goto, Kenya Kamimura, Yoko Shinagawa‐Kobayashi, Norihiro Sakai, Takuro Nagoya, Yusuke Niwa, Masayoshi Ko, Kohei Ogawa, Ryosuke Inoue, Takeshi Yokoo, Akira Sakamaki, Hiroteru Kamimura, Satoshi Abe, Hiroshi Nishina, Shuji Terai

**Affiliations:** ^1^ Division of Gastroenterology and Hepatology Graduate School of Medical and Dental Sciences Niigata University Japan; ^2^ Department of Developmental and Regenerative Biology Medical Research Institute Tokyo Medical and Dental University Japan

**Keywords:** d‐rR/Tokyo medaka, liver fibrosis, NASH, SGLT2, tofogliflozin

## Abstract

The rise in the incidence of nonalcoholic steatohepatitis (NASH) has necessitated the development of an effective prevention methodology. An antidiabetic drug, belonging to the group of sodium glucose cotransporter 2 (SGLT2) inhibitors, has been tested for its therapeutic effect on NASH; however, no studies to date have demonstrated the preventive effect of an SGLT2 inhibitor on the histological progression of steatosis and fibrosis in a sequential manner in animal models. In the present study, we examined the effect of the SGLT2 inhibitor, tofogliflozin (Tofo), on NASH liver tissue using medaka as an animal model, maintaining a feeding amount and drug concentration in all animal bodies. We generated a medaka NASH model by feeding d‐rR/Tokyo medaka a high‐fat diet and administered Tofo by dissolving the drug directly in the water of the feeding tank. Thereafter, the effects of Tofo on body weight (BW), liver weight, hepatotoxicity, fatty infiltration, and fibrotic changes in the liver were examined. We report here that SGLT2 is expressed in medaka fish and that Tofo inhibits the accumulation of fatty tissue and delays the progression of liver fibrosis in the medaka NASH model by inhibiting increases in blood sugar, serum lipids, and transaminase, irrespective of changes in BW. These results suggest that Tofo is effective for treating NASH and that the medaka model may be useful for developing new therapeutic drugs for this disease.

**Abbreviations**
ALT: alanine transaminase
HE: hematoxylin and eosin
HFD: high‐fat diet
NAFLD: nonalcoholic fatty liver disease
NAFL: nonalcoholic fatty liver
NASH: nonalcoholic steatohepatitis
SGLT2I: sodium glucose cotransporter 2 inhibitor
SGLT2: sodium glucose cotransporter 2
TC: total cholesterol
TG: triglyceride
Tofo: tofogliflozin

Nonalcoholic fatty liver disease (NAFLD) is closely associated with obesity, diabetes, hyperlipidemia, hypertension, and insulin resistance 1, 2, 3, the disease spectrum ranging from nonalcoholic fatty liver (NAFL) to nonalcoholic steatohepatitis (NASH), cirrhosis, and hepatocellular carcinoma 4, 5, 6. Insulin resistance is a major risk factor for NASH progression; therefore, a variety of antidiabetic therapies are expected to prevent NAFLD progression 7, 8, 9. Among them, sodium glucose cotransporter 2 inhibitor (SGLT2I) is a new class of oral diabetic medications that reduces hyperglycemia by suppressing the reabsorption of glucose in the proximal tubules and improving insulin resistance, glucotoxicity, and lipotoxicity 10, 11, 12. Therefore, SGLT2Is are believed to contribute to NASH management 6, 12. While clinical cases have shown the possible efficacy of SGLT2Is in NASH in terms of decrease in the serum transaminase level, body weight (BW), FIB‐4 index, HbA1c, and abdominal fat area 13, 14, 15, 16, 17, 18, 19, 20, 21, 22, 23, 24, 25, 26, direct evidence of the prevention of hepatic steatosis and progression to fibrosis in a sequential manner in NASH animal models has not been demonstrated. This is partly caused by the lack of an ideal animal model that can maintain the drug concentration in the body because the murine models showed differences in food intake and drug dosing owing to hunger induced by SGLT2Is, leading to inconsistent results in each study 13, 17, 27.

Therefore, in this study, we used medaka NASH model to examine the effect of tofogliflozin (Tofo), a highly specific SGLT2 inhibitor 23, on fatty infiltration and the fibrotic changes in the liver along with BW, liver weight (LW), and hepatotoxicity. Medaka (*Oryzias latipes*) is one of the most useful aquatic experimental animals for testing the effect and safety of drugs because of the ease of maintenance of the animal bodies in the same condition (same concentration of chemicals in the water), and high fecundity. Medaka disease models, including the NASH model, are proven to be useful 28, 29.

Our study demonstrated Sglt2 expression in medaka kidney and showed that steatosis and fibrosis were ameliorated, irrespective of the changes in the BW in medaka NASH models treated with Tofo. Our results suggest an anti‐NASH effect of a SGLT2I drug and have provided a novel option for this NASH animal model to contribute further to the development of new therapeutic drugs.

## Materials and methods

### Animals and diets

All the animal experiments were conducted in full compliance with the regulations of the Institutional Animal Care and Use Committee at Niigata University (Niigata, Japan) that approved the study protocol. All the animals received humane care as per the criteria outlined in the ‘Guide for the Care and Use of Laboratory Animals’ prepared by the National Academy of Sciences (USA) and published by the National Institute of Health (NIH publication 86‐23, revised 1985). d‐rR/Tokyo (Strain ID: MT837) was supplied by NBRP Medaka (https://shigen.nig.ac.jp/medaka/). Fish used in the experiments were 4 months old. The fish were kept in 2 L of tap water in plastic tanks under fluorescent light from 8 AM to 8 PM. The water temperature was maintained at 25 ± 1 °C. The medaka NASH model was developed by feeding the medaka a high‐fat diet (HFD) using the previously reported method 28. Briefly, each tank was supplied with a control diet or a HFD at 20 mg per fish daily, with all the provided food being consumed within 14 h. The energy content of the control diet was 3.8 kcal·g^−1^, with 23.2% of the calories coming from fat, 44.0% coming from protein, and 32.7% coming from carbohydrate; vitamins and minerals were provided as recommended (Hikari Labo M‐450; Kyorin Co. Ltd, Hyogo, Japan). The energy content of the HFD was 5.1 kcal·g^−1^, with 56.7% of calories from fat, 20.1% from protein, and 23.2% from carbohydrates; vitamins and minerals were added as recommended (HFD32; CLEA Japan, Tokyo, Japan). To confirm the successful development of medaka NASH model, chow‐diet‐fed medaka (*n* = 5–6 at each time point) were confirmed for their BW, LW/BW ratio, fat deposition area in the liver, fibrotic area in the liver, and blood sugar (Table S1).

Every 5–6 medakas at each of the four time points for the HFD and HFD + Tofo groups were prepared and analyzed, that is, > 20 medakas for each time point. To confirm the results, each experiment was repeated thrice; therefore, in total, ~ 80 medakas were assessed for the HFD and HFD + Tofo groups.

### Tofogliflozin administration

Tofogliflozin (Kowa Co. Ltd., Tokyo, Japan) has a higher solubility in dimethyl sulfoxide; therefore, although it also is stable in water, to maintain the concentration of the Tofo in the water in tanks, it was dissolved in dimethyl sulfoxide (Nacalai Tesque, Kyoto, Japan) to a concentration of 100 mg·mL^−1^ before administration to the test tank of the Tofo group at a final concentration of 0.5 mg·L^−1^, the same concentration with a *C*_max_ of 500 ng·mL^−1^ in humans treated with the standard dose of 20 mg Tofo. This determination of the concentration in the tank is consistent with our previous report using telmisartan in a medaka model 30. The same amount of dimethyl sulfoxide was administered to the tank of the HFD group. The water, HFD, and Tofo in the tank were exchanged every 2 days, and tanks were carefully washed to maintain a consistent concentration.

### Histological analyses

Liver tissue samples were collected at the appropriate time points, fixed in 10% formalin, and embedded in paraffin. Sections (10 μm) were stained with standard hematoxylin and eosin (HE) and Sirius Red. Hepatocyte ballooning and fat deposition in the liver were detected by HE staining, and fibrotic tissue in the liver was detected as the area stained red by Sirius Red staining. Then, the images were captured from each tissue section randomly, and a quantitative analysis of fat deposition areas and fibrotic areas was performed using the imagej software (version 1.6.0_20; National Institutes of Health, Bethesda, MD, USA) with RGB‐based protocol as reported previously 31. Rabbit anti‐SGLT2 polyclonal antibody (ab85626, 1 : 200 dilution; Abcam, Cambridge, MA, USA), Vectastain Elite ABC Rabbit IgG kit (PK‐6101; Vector Laboratories, Burlingame, CA, USA), and DAB chromogen tablet (Muto Pure Chemicals, Tokyo, Japan) were used for the immunohistochemical (IHC) staining.

### Biochemical analyses

Blood samples were collected from the medaka after 12‐h fasting for all collections. Fish were kept on ice for 1 min; thereafter, they were bled by cutting the ventral portion of the tail fin. Blood was collected in a heparinized microcapillary tube (VC‐H075H; Terumo, Tokyo, Japan) and centrifuged at 1200 ***g*** for 12 min at 22 °C. The biochemical analyses of plasma total cholesterol (TC), triglyceride (TG), and alanine transaminase (ALT) were performed in Oriental Yeast Co. Ltd. (Shiga, Japan). For biochemical assays, more than 40 μL of blood is necessary, and the total blood volume that can be collected from a medaka is ~ 2 μL; therefore, blood samples from 20 animals from the three repeated experiments were pooled and measured at once as an averaged value. A glucometer (Glucocard G Black, GT‐1830; Arkray Co. Ltd, Kyoto, Japan) requiring < 2 μL of blood volume was used to determine the blood sugar concentration.

### Statistical analyses

The obtained data were analyzed using either Student's *t*‐test or a two‐way factor repeated‐measures analysis of variance (ANOVA) followed by a Bonferroni's multiple comparison test. A *P* value ≤ 0.05 was considered to indicate statistical significance.

## Results

### Sglt2 expression in the medaka model

**Figure 1:**
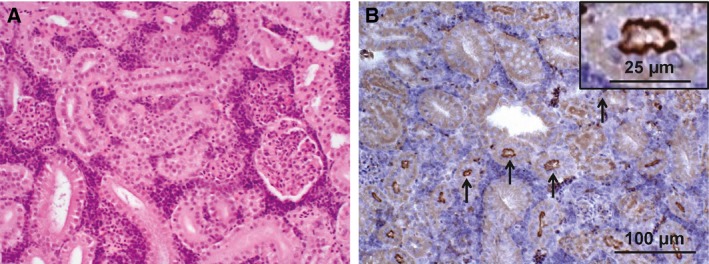
Expression of Sglt2 in the medaka kidney. (A) HE staining of the medaka kidney. (B) IHC staining of the medaka kidney using anti‐SGLT2 antibody. Black arrows indicate the positively stained proximal tubular cells in the medaka kidney. The scale bar represents 100 μm, and the scale bar in the inset represents 25 μm.

As previously reported, *Sglt2* is conserved in invertebrates and vertebrates containing 13 exons 32, and various fishes, including *Squalus acanthias* 33, *Leucoraja erinacea* 34, and *Carassius auratus* 35, also express this gene with ~ 65% homology with that of humans. Its function also is reported to be similar to that of humans 35. Therefore, to confirm and determine Sglt2 protein expression in medaka and to detect with available antibodies, we confirmed the Sglt2 genetic sequence using genomic DNA collected from fin tissues. Medaka Sglt2 shows about 69.1% protein sequence homology with human and full homology in the C terminus (data not shown). IHC analyses using the antibody directed against Sglt2 C terminus confirmed the expression of Sglt2 protein in the proximal tubules of the medaka kidneys. Figure 1 shows the histology of the medaka kidney with its basic structure similar to that of other species (Fig. 1a). IHC shows positive staining of the luminal surface of the proximal tubular cells similar to the Sglt2 antibody staining in other animal species (Fig. 1b). Thus, Sglt2 protein is expressed in the luminal surface of the proximal tubular cells in the medaka kidney, and the medaka animal model can be used to examine the effect of the SGLT2 inhibitor.

### Effects of Tofo on medaka NASH model

#### Effects of Tofo on the macroscopic findings and the body and liver weights of medaka NASH model

**Figure 2:**
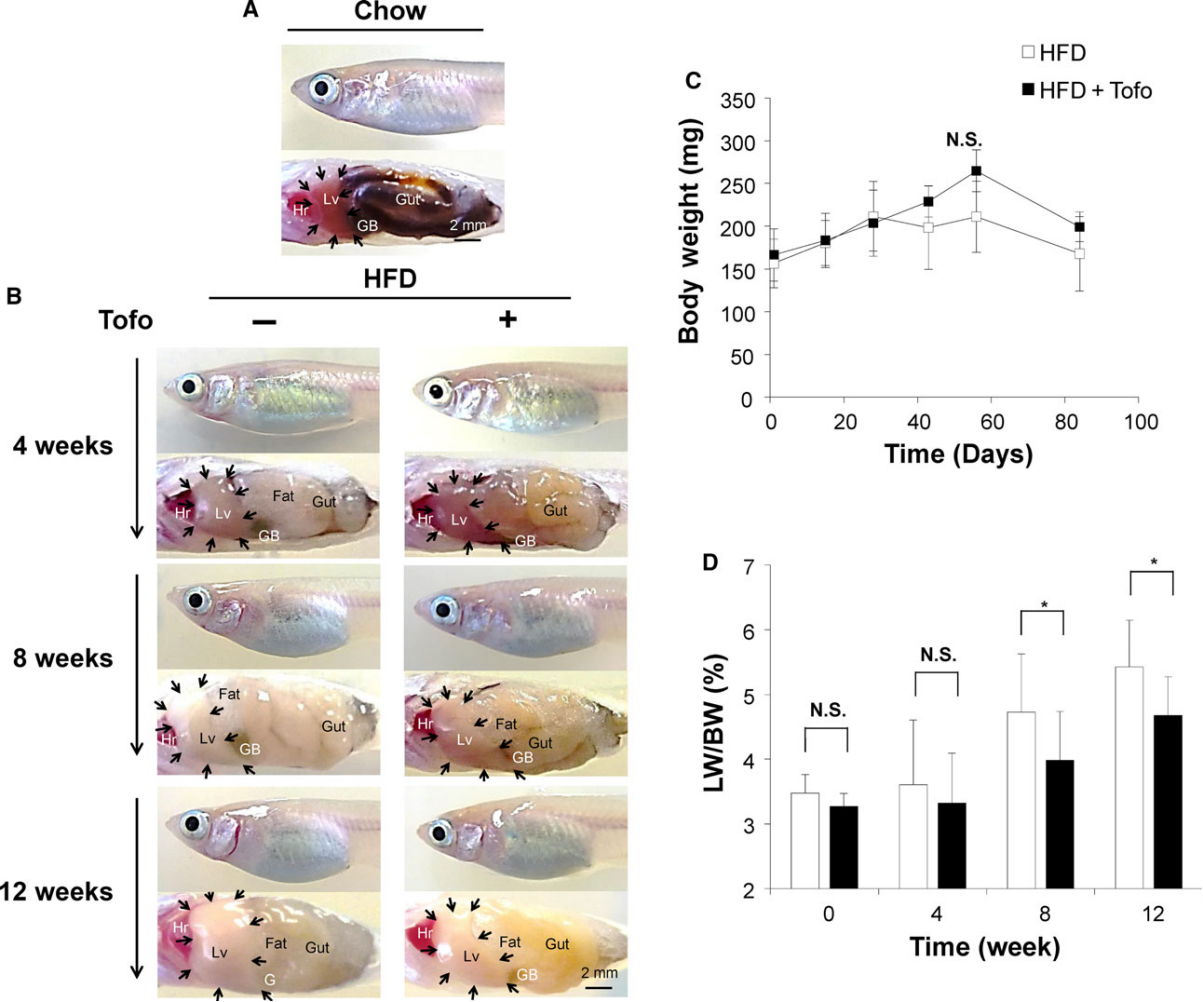
Effects of Tofo on the macroscopic findings and the body and LWs of medaka NASH model. Representative macroscopic appearance of medaka. (A) Appearance of chow‐diet‐fed medaka. (B) Appearance of medaka fed with HFD and HFD + Tofo for 4, 8, and 12 weeks, respectively. Hr, heart; Lv, liver; GB, gallbladder; Gut, digestive tract. Black arrows indicate the edge of the liver in each model. Scale bar represents 2 mm. LW and BW were calculated at the appropriate time points. (C) Change in BW. The values represent mean ± SD (*n* = 15 for each group). N.S., no statistical significance. Two‐way ANOVA followed by Bonferroni's multiple comparison test. (D) The ratio of LW/BW. The values represent mean ± SD (*n* = 15 for each group). *P <* *0.05, and N.S., no statistical significance. Student's *t*‐test.

The medaka NASH model was developed using HFD as previously reported 28 to investigate the effect of Tofo on the model. Time‐dependent macroscopic changes in the liver were assessed by comparing the livers of HFD‐fed medaka treated or untreated with Tofo for 12 weeks and comparing them to controls on normal chow (Fig. 2). Medaka fed with HFD showed a time‐dependent enlargement of the liver with the development of a whitish color compared with the liver of chow‐diet‐fed medaka (Fig. 2a,b) consistent with our previously reported results 28, and Tofo‐treated medaka showed delayed liver swelling and whitish change (Fig. 2b). With the increase of visceral fat tissue, the gut of HFD‐fed medaka was covered by fatty tissue (Fig. 2a). The LW and BW of HFD‐fed NASH medaka were examined, and the ratio of LW/BW was assessed to quantify the effect of Tofo on the LW. There was no significant difference in the BW among HFD‐fed medaka and HFD + Tofo‐fed medaka (Fig. 2c). The LW/BW ratio was 3.60 ± 1.00% and 3.32 ± 0.77% in the HFD‐fed medaka and HFD + Tofo‐fed medaka, respectively, showing no significant difference at 4 weeks (Fig. 2d). At 8 weeks, HFD + Tofo‐fed medaka showed a significantly (*P* < 0.05) lower LW/BW ratio of 3.98 ± 0.76% as compared to that in HFD‐fed medaka for which the ratio was 4.73 ± 0.90%. At 12 weeks, a significant difference persisted, with a LW/BW ratio of 4.68 ± 0.60% in the HFD + Tofo‐fed medaka being significantly lower than that in the HFD‐fed medaka (5.43 ± 0.72%, *P* < 0.05; Fig. 2d). These results indicate that Tofo is effective in preventing liver enlargement and swelling caused by HFD.

#### Effects of Tofo on hepatocyte ballooning and fat deposition in the liver of NASH medaka

**Figure 3:**
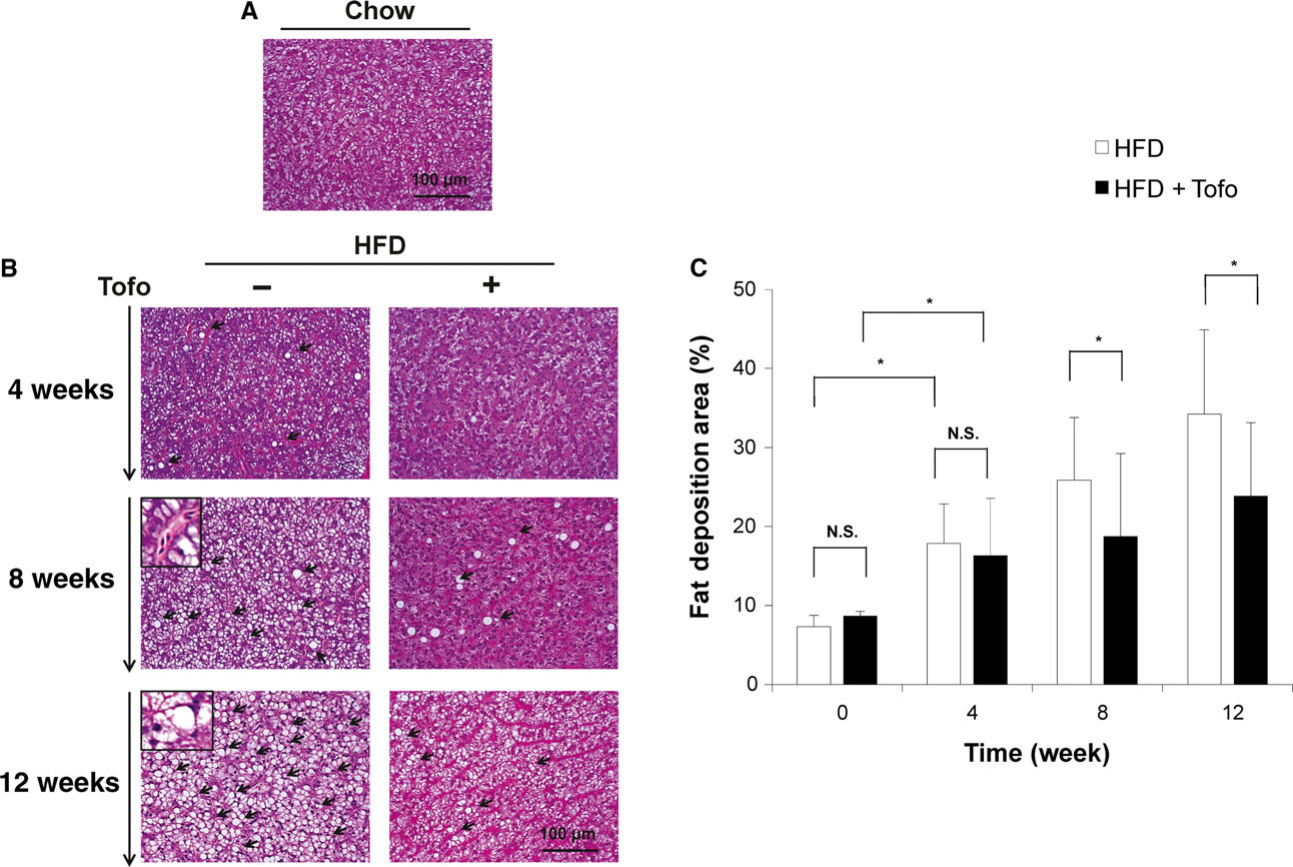
Effects of Tofo on the histological changes and the deposition of fatty tissue in the liver of medaka NASH model. Representative microscopic findings of medaka liver tissues stained with HE. (A) Chow‐diet‐fed medaka. (B) Medaka fed with HFD and HFD + Tofo for 4, 8, and 12 weeks. The insets in HFD for 8 and 12 weeks indicate the inflammatory cells and ballooning degeneration of the hepatocyte, respectively. Black arrows indicate fat deposition areas. Scale bar represents 100 μm. (C) Quantitative analysis of fat deposition areas in the medaka liver. The values represent mean ± SD (*n* = 15 for each group). *P <* *0.05, and N.S., no statistical significance. Student's *t*‐test.

Hematoxylin and eosin staining was performed to determine the histological changes in the liver of HFD‐fed medaka treated or untreated with Tofo for 12 weeks and compared to controls on normal chow (Fig. 3). Representative histological analyses of medaka fed with a normal diet are shown in Fig. 3a and of those fed HFD for 4, 8, and 12 weeks and HFD + Tofo for 4, 8, and 12 weeks are shown in Fig. 3b. Medaka fed with HFD showed a time‐dependent accumulation of fat tissue compared to the liver of chow‐diet‐fed medaka (Fig. 3a,b) consistent with our previously reported results 28. Additionally, the HFD group exhibited more accumulation of the inflammatory cells and ballooning degeneration of the hepatocyte and macrovesicular fat deposition in a liver tissue in a time‐dependent manner (Fig. 3b inset) compared to those in the NASH medaka treated with Tofo. The fatty changed areas included in these models were assessed via quantitative analyses (Fig. 3c). The deposition of fat in the liver tissue in both the HFD‐fed and HFD + Tofo‐fed medaka groups (Fig. 3b) was significantly higher (17.83 ± 5.03% and 16.30 ± 7.29%, respectively, no difference) than that of the control group fed normal chow (7.36 ± 3.28%; *P* < 0.01) at 4 weeks (Fig. 3c). At 8 weeks after Tofo treatment, a significant difference between the HFD‐fed and HFD + Tofo‐fed medaka groups was clear (Fig. 3b) with 25.89 ± 7.90% and 18.74 ± 10.51% of fatty tissues, respectively. Further 4 weeks of Tofo treatment produced a fat area of 23.83 ± 9.33%, significantly lower than the area observed in non‐Tofo‐treated medaka on HFD (34.25 ± 10.67%, *P* < 0.05; Fig. 3c). These results show that Tofo has an effect on the HFD‐induced medaka NASH model, delaying the accumulation of fat tissues in the liver.

#### Effects of Tofo on the liver fibrotic tissue of NASH medaka

**Figure 4:**
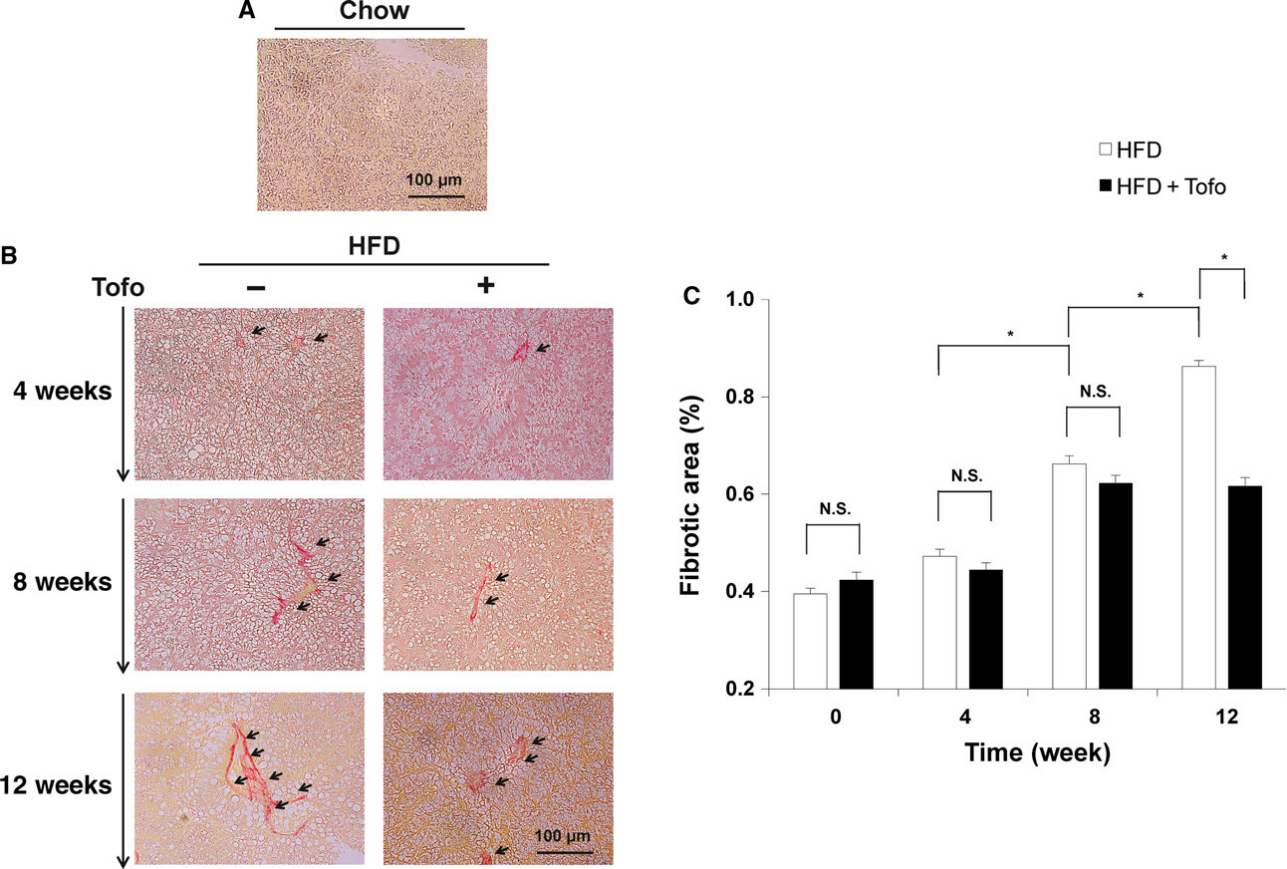
Effects of Tofo on the deposition of fibrotic tissue in the liver of medaka NASH model. Representative microscopic findings of medaka liver tissues stained with Sirius Red. (A) Chow‐diet‐fed medaka. (B) Medaka fed with HFD and HFD + Tofo for 4, 8, and 12 weeks. Black arrows indicate fibrotic tissues. Scale bar represents 100 μm. (C) Quantitative analysis of the fibrotic area in the medaka liver. The values represent mean ± SD (*n* = 15 for each group). *P <* *0.05, and N.S., no statistical significance. Student's *t*‐test.

Sirius Red staining was performed to determine histological liver fibrotic changes in the liver of HFD‐fed medaka treated or untreated with Tofo for 12 weeks and compared with those fed normal chow as controls (Fig. 4). Representative histological analyses of medaka fed a normal diet are presented in Fig. 4a and of those fed HFD for 4, 8, and 12 weeks and HFD + Tofo for 4, 8, and 12 weeks are shown in Fig. 4b. Medaka fed with HFD showed a time‐dependent increase in fibrotic tissue compared with the liver of chow‐diet‐fed medaka (Fig. 4a,b). Additionally, the HFD group exhibited more fibrotic tissue than the HFD group treated with Tofo 12 weeks after administration. The quantitative analyses of fat areas in these models were assessed to examine the effect (Fig. 4c). The fibrotic liver tissue in both the HFD and HFD + Tofo‐fed medaka groups showed a time‐dependent increase although the HFD + Tofo group had significantly lower fibrosis levels than the HFD‐fed medaka at 12 weeks after treatment (0.86 ± 0.01% versus 0.62 ± 0.02%, respectively; Fig. 4c). These results show that Tofo has an effect on the HFD‐induced medaka NASH model, delaying the progression of liver fibrosis.

#### Effects of Tofo on biochemical analyses

**Figure 5:**
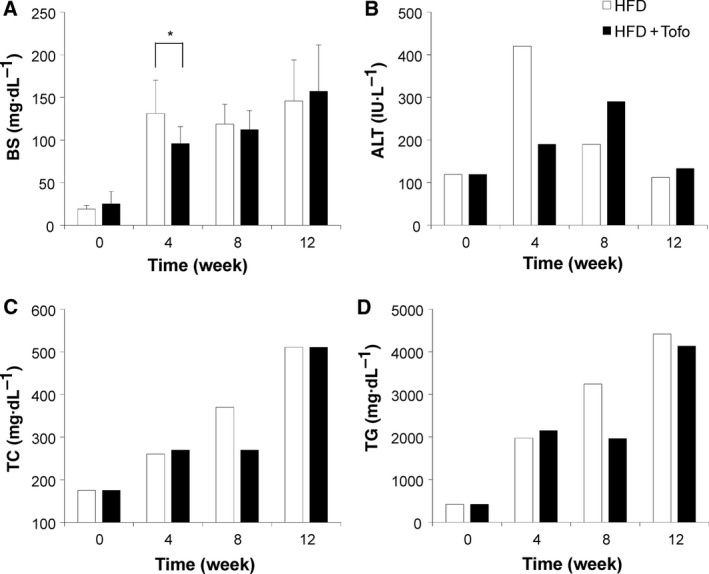
Effects of Tofo on the serum biochemical markers. The time‐dependent changes in the serum biochemical markers. The values represent average concentrations of (A) BS; (B) alanine aminotransferase (ALT); (C) TC; and (D) TG. The values represent mean ± SD (*n* = 20 for each group). *P <* *0.05, and N.S., no statistical significance. Student's *t*‐test.

To examine the effect of Tofo on serum biochemical markers, time‐dependent biochemical analyses were performed (Fig. 5). Plasma glucose was increased significantly from a control level of 18.9 ± 4.4 to 131.3 ± 39.1 mg·dL^−1^ in HFD‐fed medaka; further, the increase was suppressed to 96.0 ± 19.7 mg·dL^−1^ in the HFD + Tofo group (*P* < 0.05). At this concentration, the effect of Tofo weakened within 8 weeks, showing similar levels in both the HFD and HFD + Tofo‐fed medaka (118.8 ± 23.4 and 112.3 ± 22.3 mg·dL^−1^, respectively), and no difference was observed at 12 weeks (146.0 ± 48.1 mg·dL^−1^ in the HFD‐fed medaka and 157.3 ± 54.2 mg·dL^−1^ in the HFD + Tofo‐fed medaka; Fig. 5a). The serum ALT level was the highest after 4 weeks in the HFD‐fed medaka (420 mg·dL^−1^) compared to that in the HFD + Tofo‐fed medaka (190 mg·dL^−1^) and in the controls (119 mg·dL^−1^). The slow decreases in the HFD‐fed medaka of 190 and 112 mg·dL^−1^ at 8 and 12 weeks, respectively, indicate a decrease in the normal hepatocytes that release the transaminase on liver injury, similar to the ‘burnout’ pattern of hepatitis. The HFD + Tofo group showed a slow increase to 290 mg·dL^−1^ at 8 weeks, and the level started to decrease, reaching 133 mg·dL^−1^ at 12 weeks, suggesting delayed peak inflammation (Fig. 5b), as confirmed by the histological analyses. The serum concentration of TC was 1.5‐fold higher in both the HFD and HFD + Tofo groups (260 and 270 mg·dL^−1^, respectively) than in the control diet group (175 mg·dL^−1^) 4 weeks after Tofo administration (Fig. 5c). At 8 weeks, the increase in the serum TC level was delayed in the Tofo‐administered NASH medaka, showing a lower level of 270 mg·dL^−1^ compared to 370 mg·dL^−1^ in the HFD‐fed medaka. This difference disappeared after further 4 weeks in the nontreated (510 mg·dL^−1^) as compared to that in the Tofo‐treated (511 mg·dL^−1^) HFD‐fed medaka (Fig. 5c). The serum levels of TG showed a similar pattern. Both HFD (1970 mg·dL^−1^) and HFD + Tofo‐fed medaka (2150 mg·dL^−1^) showed a significant increase in the TG as compared to that in the normal controls (420 mg·dL^−1^) at 4 weeks, confirming the successful development of an animal model for NASH. While 4‐week Tofo administration had no effect on TG levels of HFD‐fed medaka (Fig. 5d), Tofo administration became effective in preventing TG increase at 8 weeks, with 3240 mg·dL^−1^ TG in HFD‐fed medaka and 1960 mg·dL^−1^ in HFD + Tofo‐fed medaka (Fig. 5d). This difference also disappeared in the subsequent 4 weeks with 4417 mg·dL^−1^ TG in HFD‐fed medaka and 4130 mg·dL^−1^ in HFD + Tofo‐fed medaka, exhibiting a similar pattern with TC. These results show that Tofo has an effect on the HFD‐induced medaka NASH model, decreasing the hepatotoxicity by reducing the glucotoxicity and lipotoxicity.

## Discussion

The development of an effective prevention methodology for NAFLD is essential, considering the rising incidence of the disease 1, 2, 3. Disease progression is related to obesity, diabetes, and insulin resistance. Therefore, SGLT2Is are believed to contribute to NASH management 12; however, the changes in liver histology over the course of treatment have been inconclusive in the murine model because SGLT2Is stimulate appetite, leading the mice to cope differently and exerting a different effect on the changes in BW; these may explain the individual differences in liver histology 13, 17, 19, 27. Therefore, in this study, using a medaka NASH model, we investigated the effect of the Tofo, which has a higher specificity in inhibiting SGLT2 23, on the NASH liver to obtain new insights on the time‐dependent effects of Tofo treatment and extensive quantitative changes in the liver histology and disease parameters, because in this model, we could maintain the serum concentration of the medicine by keeping them in the water of the averaged concentrated with medicines.

After demonstrating the Sglt2 expression in medaka, we showed that Tofo inhibited the accumulation of fatty tissue and the progression of liver fibrosis and toxicity. In addition, these changes were independent of the change in BW, indicating that improvement in insulin resistance is key in NASH treatment. Our study seemed to have a limitation in BS analysis because SGLT2I increased the sugar secretion from the body into the urine and medakas are kept in the water with their urine, which could affect the analyses. However, because the urinary volume of medaka has been reported to be mL·h^−1^·kg^−1^ in a fish 36 and we exchanged the water, HFD, and Tofo in the tank every 2 days, the urine volume of 10 medakas of ~ 200 mg of 0.1 mL in a tank filled with 2 L water was low enough, which is unable to be detected by Oriental Yeast Co. Ltd. or a glucometer, and therefore, can be ignored for sugar concentration analyses. In addition, our experiment showed that the sugar concentration was still below the detection limit with the glucometer even accumulating 20 medakas in one tank, probably due to the small volume of urine; therefore, further study to sample the small amount of urine directly by the *in situ* micropuncture technique from medaka kidney will show the minute time‐dependent change in urinary sugar level.

In conclusion, we showed Sglt2 expression in medaka kidney and found that the highly specific SGLT2I, Tofo, prevents NASH progression by preventing steatosis and fibrosis. These results suggest that SGLT2I is a promising treatment option for NASH and that the medaka model could be useful for testing SGLT2Is and more chemical compounds for their efficacy on NASH in a cost‐efficient manner.

## Conflict of interest

The authors declare no conflict of interest.

## Author contributions

RG, YSK, NS, TN, YN, MK, KO, and RI performed experiments; KK, TY, AS, HK, and SA analyzed the data; KK, HN, and ST conceived and supervised the study; and KK and ST wrote the manuscript.

## Supporting information


**Table S1**. The data set of chow‐diet‐fed control medaka.
