# Supplementary figures and images for: Inhibition of sodium glucose cotransporter 2 (SGLT2) delays liver fibrosis in a medaka model of nonalcoholic steatohepatitis (NASH)
Source: FEBS Open Bio. 2019 Feb 15;9(4):643–52. doi: 10.1002/2211-5463.12598 (PMC6443870; doi:10.1002/2211-5463.12598)

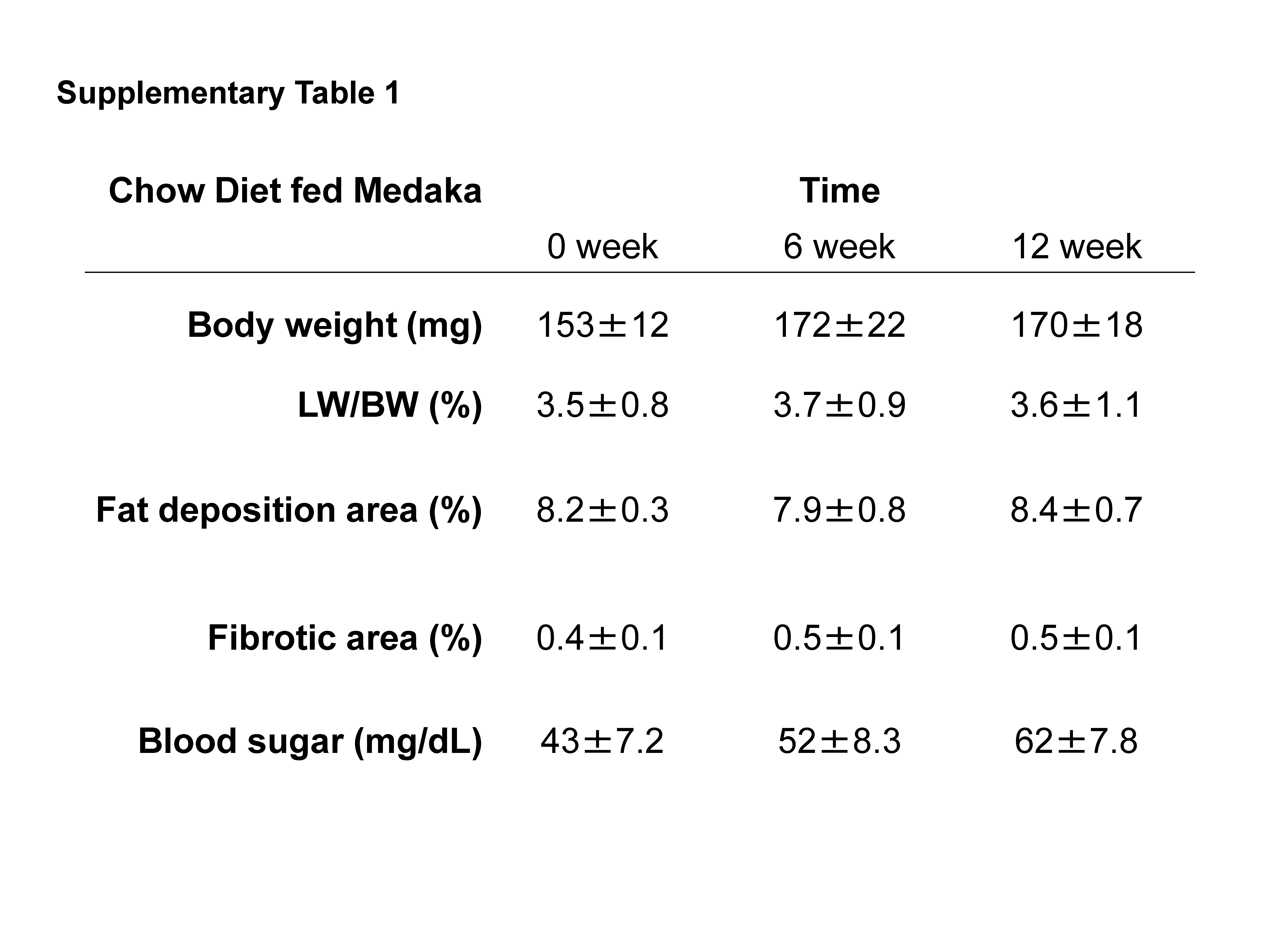

Supplement: Supplementary file 1 — Table S1. The data set of chow‐diet‐fed control medaka. [file FEB4-9-643-s001.tiff]
